# Supplementary material for: Vocational rehabilitation for people with multiple sclerosis in the national health service of the United Kingdom: A realist evaluation
Source: PLoS One. 2025 Feb 25;20(2):e0319287. doi: 10.1371/journal.pone.0319287 (PMC11856266; doi:10.1371/journal.pone.0319287)
Supplement: S2 File — (DOCX) [file pone.0319287.s002.docx]

**Supplementary Information 2- Search Strategy**

**PubMed**

("Multiple Sclerosis"[mh] OR "Myelitis, Transverse"[mh:noexp] OR "Demyelinating Diseases"[mh:noexp] OR "Encephalomyelitis, Acute Disseminated"[mh:noexp] OR "Optic Neuritis"[mh] OR "multiple sclerosis" OR "neuromyelitis optica" OR "transverse myelitis" OR encephalomyelitis OR devic OR "optic neuritis" OR "demyelinating disease*" OR "acute disseminated encephalomyelitis") AND ("Rehabilitation, Vocational" [Mesh] OR "Employment"[Mesh] OR "Employment, Supported" [Mesh] OR "Work capacity evaluation" [Mesh] OR “Sick leave” [Mesh] OR “work retention” OR “job retention” OR “vocational assessment” OR “vocational education” OR “vocational guidance” OR “work* modification*” OR “sheltered workshop” OR “career mobility” OR “absenteeism” OR “presenteeism”)

**MEDLINE (OVID)**

#1 Exp Multiple Sclerosis/

#2 Myelitis, Transverse/

#3 Demyelinating Diseases/

#4 Encephalomyelitis, Acute Disseminated/

#5 exp Optic Neuritis/

#6 neuromyelitis optica/

#7 encephalomyelitis/

#8 1 or 2 or 3 or 4 or 5 or 6 or 7

#9 exp Rehabilitation, Vocational/

#10 exp Employment/

#11 exp Employment, Supported/

#12 exp Work Capacity Evaluation/

#13 Sick Leave.mp.

#14 exp Work/

#15 work retention.mp.

#16 job retention.mp.

#17 vocational assessment.mp.

#18 exp Vocational Education/

#19 vocational guidance.mp.

#20 Work* modification*.mp.

#21 Work* adjustment*.mp.

#22 sheltered workshop*.mp.

#23 career mobility.mp.

#24 9 or 10 or 11 or 12 or 13 or 14 or 15 or 16 or 17 or 18 or 19 or 20 or 21 or 22 or 23

#25 #8 AND #24

**EMBASE (OVID)**

#1 Exp Multiple Sclerosis/

#2 Myelitis, Transverse/

#3 Demyelinating Diseases/

#4 Encephalomyelitis, Acute Disseminated/

#5 exp Optic Neuritis/

#6 neuromyelitis optica/

#7 encephalomyelitis/

#8 1 or 2 or 3 or 4 or 5 or 6 or 7

#9 exp Rehabilitation, Vocational/

#10 exp Employment/

#11 exp Employment, Supported/

#12 exp Work Capacity Evaluation/

#13 Sick Leave.mp.

#14 exp Work/

#15 work retention.mp.

#16 job retention.mp.

#17 vocational assessment.mp.

#18 exp Vocational Education/

#19 vocational guidance.mp.

#20 Work* modification*.mp.

#21 Work* adjustment*.mp.

#22 sheltered workshop*.mp.

#23 career mobility.mp.

#24 9 or 10 or 11 or 12 or 13 or 14 or 15 or 16 or 17 or 18 or 19 or 20 or 21 or 22 or 23

#25 #8 AND #24

**Web of Science**

#1 Multiple Sclerosis

#2 Myelitis, Transverse

#3 Demyelinating Diseases

#4 Encephalomyelitis Acute Disseminated

#5 “Optic Neuritis”

#6 neuromyelitis optica

#7 encephalomyelitis

#8 1 or 2 or 3 or 4 or 5 or 6 or 7

#9 Vocational Rehabilitation

#10 Employment

#11 Supported employment

#12 Work Capacity Evaluation

#13 Sick leave

#14 work retention

#15 job retention

#16 Work modification

#17 Work adjustment

#18 vocational assessment

#19 vocational education

#20 vocational guidance

#21 sheltered workshop

#22 career mobility

#23 9 or 10 or 11 or 12 or 13 or 14 or 15 or 16 or 17 or 18 or 19 or 20 or 21 or 22

#24 8 and 23

**PsycInfo (OVID)**

#1 Exp Multiple Sclerosis/

#2 transverse myelitis.mp.

#3 Demyelinating Diseases.mp.

#4 encephalomyelitis/

#5 exp Optic Neuritis/

#6 neuromyelitis optica.mp.

#7 1 or 2 or 3 or 4 or 5 or 6

#8 exp Rehabilitation, Vocational/

#9 exp Employment/

#10 exp Supported Employment/

#11 work capacity evaluation.mp.

#12 Sick Leave.mp.

#13 Exp Employee Retention/

#14 job retention.mp.

#15 vocational assessment.mp.

#16 exp Vocational Education/

#17 vocational guidance.mp.

#18 Work* modification*.mp.

#19 Work* adjustment*.mp.

#20 sheltered workshop*.mp.

#21 career mobility.mp.

#22 8 or 9 or 10 or 11 or 12 or 13 or 14 or 15 or 16 or 17 or 18 or 19 or 20 or 21

#23 7 and 22

**CINAHL**

((((MH multiple sclerosis) OR (MH Myelitis, Transverse) OR (MH optic neuritis) OR (MH Encephalomyelitis, Acute Disseminated) OR (MM “neuromyelitis optica”) OR (MM “myelitis transverse”) OR (MH “Demyelinating Diseases+”))) OR ((((MM “encephalomyelitis”) AND
(MH "Rehabilitation, Vocational") OR (MH "Work+") OR (MH "Employment+") OR (MH "Employment, Supported+") OR (MH "Work Capacity Evaluation+") OR (MM "Work Capacity Evaluation") OR (work retention OR job retention) OR (vocational assessment OR MM "vocational education" OR vocational guidance) OR (MM “job accommodation”) OR (MM "sheltered workshop") OR (MM "career mobility")))

**Google Scholar**

“Vocational rehabilitation” AND “multiple sclerosis” AND “NHS”

**British Library**

“Vocational rehabilitation” AND “multiple sclerosis”

**Ethos**

“Vocational rehabilitation” AND “multiple sclerosis”

**Clinical Trial.gov**

“Vocational rehabilitation” AND “multiple sclerosis”
