# Supplementary material for: Vocational rehabilitation for people with multiple sclerosis in the national health service of the United Kingdom: A realist evaluation
Source: PLoS One. 2025 Feb 25;20(2):e0319287. doi: 10.1371/journal.pone.0319287 (PMC11856266; doi:10.1371/journal.pone.0319287)
Supplement: S3 File — (DOCX) [file pone.0319287.s003.docx]

## **Supplementary Information 3– Data Extraction Form and Quality Assessment**

Data Extraction Tool

**Full Study Reference:**

**Author’s email address:**

**Extracted from Search number:**

| **Data Extractor Initials** |  | **VR Programme Name** |  | **Setting** |  |
| --- | --- | --- | --- | --- | --- |

| **References Extracted from Manuscript**  (Add APA Style reference & DOI) |
| --- |
|  |
|  |
|  |
|  |
|  |

**Relevance**

| **Does the document include any data that might be relevant to our programme theories? Which ones?** | |
| --- | --- |
| **Programme Theories** | **Reasoning- Why this paper is talking about this programme theory? To what context and outcome are these mechanisms linked to?** |
| Early intervention |  |
| Individually tailored |  |
| Employer engagement |  |
| Providing reasonable accommodations |  |
| Collaboration/ Coordinated effort |  |
| Health Care Professional (NHS) involvement |  |
| Other: |  |

**STUDY SPECIFIC CMOs IDENTIFIED/REPORTED**

Is there any evidence in the text that provides data that can be interpreted as a Context, mechanism (resource/response) or outcome? What are the CMOs [Context-Mechanism (resource)-Mechanism (response)-Outcome Configurations (CMOC)] for the data?

| **Context** | **Resource/**  **Intervention** | **Response/**  **Mechanism** | **Outcome** | **Mechanism Level** | **Health Condition** | **Full/**  **Partial** |
| --- | --- | --- | --- | --- | --- | --- |
|  |  |  |  |  |  |  |
|  |  |  |  |  |  |  |
| **F= Full / P= Partial CMO / Mechanisms Level:** Micro = individual (PwMS + Employer + Therapist); Messo = team (implementer team, or PwMS + carer(s) as a team, or care team); Macro = organisation level (hospital, clinic, workplace). Cells Coloured in Orange: Context where the mechanism may not fire | | | | | | |

**Judgements about programme theory**

| ***What should be added from this paper to the programme theory?***   - *How does this (full or partial) CMOC relate to the programme theory?* - *Considering this CMOC and any supporting data, does the programme theory need to be changed? How?*   *Information to help me refine the programme. Justify decision of why CMOs were included***.** |
| --- |
|  |
