# Supplementary material for: Vocational rehabilitation for people with multiple sclerosis in the national health service of the United Kingdom: A realist evaluation
Source: PLoS One. 2025 Feb 25;20(2):e0319287. doi: 10.1371/journal.pone.0319287 (PMC11856266; doi:10.1371/journal.pone.0319287)
Supplement: S4 File — (DOCX) [file pone.0319287.s004.docx]

## **Supplementary Information 4- Criteria for scoring Rigor and Relevance**

**Assessment of Rigour**

| Where the methods used to generate the relevant data credible and trustworthy? | | | | | | |
| --- | --- | --- | --- | --- | --- | --- |
| **No rigour whatsoever (e.g., commentary)** | **1** | **2** | **3** | **4** | **5** | **Exceptional rigour (e.g., well designed research)** |
|  |  |  |  |  |  |  |
| **Comments and concerns about rigour** | | | | | | |
| *Justify score* | | | | | | |

To justify the score, follow the TAPUPAS criteria developed by Pawson et al. (2003) to assess issues regarding validity and rigour:

- Transparency: is the process of knowledge generation open to outside scrutiny?
- Accuracy: are the claims made based on relevant and appropriate information?
- Purposivity: are the methods used fit for the purpose?
- Utility: are the knowledge claims appropriate to the needs of the practitioner?
- Propriety: has the research been conducted ethically and legally?
- Accessibility: is the research presented in a style that is accessible to the practitioner?
- Specificity: does the knowledge generated reach source-specific standards?

| **Score** | **Reasoning** |
| --- | --- |
| **1** | The study meets **none** of the TAPUPAS criteria. |
| **2** | The study meets **one or two** of the TAPUPAS criteria. |
| **3** | The study meets **three or four** of the TAPUPAS criteria. |
| **4** | The study meets **five or six** of the TAPUPAS criteria. |
| **5** | The study meets all (**seven**) of the TAPUPAS criteria. |

**Reference**

R Pawson, A Boaz, L Grayson, A Long, & C Barnes. (2003). *Types and Quality of Knowledge in Social Care*.

**Assessment of Relevance**

| How relevant is this article to the aims of the realist review in refining or testing the programme theory? | | | | | | |
| --- | --- | --- | --- | --- | --- | --- |
| **Not relevant whatsoever** | **1** | **2** | **3** | **4** | **5** | **Extremely relevant** |
|  |  |  |  |  |  |  |
| **Comments and concerns about Relevance** | | | | | | |
| *Justify score* | | | | | | |

| **Score** | **Reasoning** |
| --- | --- |
| **1** | The document meets the inclusion criteria for the review but provides little to no information to inform any aspect of the programme theory. |
| **2** | The document provides at least some information to inform one aspect of the programme theory. |
| **3** | The document provides some information to inform at least two aspects of the programme theory. |
| **4** | The document provides extensive information to inform at least one aspect of the programme theory.  **OR**  The document provides some information about three or more aspects of the programme theory. |
| **5** | The document provides extensive information to inform two or more aspects of the programme theory. |

**Other Comment**

| **Other comments about the article** |
| --- |
|  |
