# Supplementary material for: Vocational rehabilitation for people with multiple sclerosis in the national health service of the United Kingdom: A realist evaluation
Source: PLoS One. 2025 Feb 25;20(2):e0319287. doi: 10.1371/journal.pone.0319287 (PMC11856266; doi:10.1371/journal.pone.0319287)
Supplement: S5 File — Semi-structured interview topic guide. (DOCX) [file pone.0319287.s005.docx]

## **Supplementary Information 5 - Interview Topic Guide**

**Semi-Structured Interview Topic Guide**

*[Please note: This is a semi-structured topic guide that is designed to be used flexibly with each participant. As such, the questions and prompts (presented as sub-questions) asked in each interview are likely to vary slightly.]*

**Introductory statement**

I am a researcher at the University of Nottingham working on a project that aims to understand what can be done to help people with Multiple Sclerosis (MS) to remain at work.

As you might remember from the information sheet, vocational rehabilitation (VR) is a process whereby those disadvantaged by illness or disability can be enabled to access, maintain, or return to employment or other useful occupation. We have previously developed a job retention VR intervention for people with MS and tested this with 15 people with MS and 3 employers.

Today, we are interested in understanding how to refine our understanding of how this intervention works (*present logic model*) so that we can incorporate the intervention within existing NHS services.

Your name will not be mentioned on any published documents, and any names or places you might refer to will be anonymized when transcribed. All information you provide will be used to develop our understanding and kept confidential.

**Testing Initial Rough Theory Programme**

We are interested in understanding how to refine our preliminary intervention programme theory (*present logic model*) to incorporate the intervention within existing NHS services.

The logic model includes some activities that could help someone with MS remain at work. We want to understand what impact each of these activities might have, which are the most important and whether there is anything else that we should consider too.

We have developed a series of “If, the, because” statements about how we think the intervention works. Taking these into consideration and additional ideas discussed today, we would like to understand how the NHS context may impact how the intervention works, and how it can impact employers, people with MS, and healthcare professionals.

Here are some examples:

| **If** | **Then** | **Because** |
| --- | --- | --- |
| OT and person with MS select an **individually tailored intervention** based on intervention goals and outcome of the initial assessment | The person with MS **understands the impact of MS at work** | The intervention provides **support that can be adapted** to needs and circumstances. |
| The OT delivers between **1 to 10 hours of vocational rehabilitation** over three months to the person with MS from a menu of intervention components | The person with MS will become able to **self-manage their condition at work**, leading to **increased workability** and/or **productivity** | The person with MS will **gain knowledge** about the impact of their MS at work and become **confident** to request support at work |
| The OT delivers **up to 4 hours of vocationa**l rehabilitation support over three months to the employer | The employer becomes **better informed about how to support the employee with MS at work** | The employer gains **insight** about the difficulties the employee with MS experiences and an **improved awareness** of how to support the employee |
| Both the person with MS and the OT **identify barriers to job retention and reasonable accommodations** to overcome the barriers | The person with MS will **receive reasonable accommodations** at work | The person with MS is **confident** to express their needs at work and how the accommodations will benefit their performance |

1. If you could change something about this programme to make it work better within the NHS, what would you change?
   1. Why?
2. What else do we need to know to understand how this programme will work within the NHS?
   1. What aspects of the programme do you think could have the greatest impact? Why?
   2. Could there be any unintended or negative consequences of [proposed activity]?
3. We could also develop new statements following the questions:
   1. IF “(proposed activity)” is provided, THEN what should be the result for the participants?
   2. WHY do you believe the activity would lead to these outcomes? On what do you base your assumption of change? Is it an established theory?
   3. Are you aware of any evidence that the activity will lead to the result? (Knowledge from literature or own research programmes)
