# Supplementary material for: Vocational rehabilitation for people with multiple sclerosis in the national health service of the United Kingdom: A realist evaluation
Source: PLoS One. 2025 Feb 25;20(2):e0319287. doi: 10.1371/journal.pone.0319287 (PMC11856266; doi:10.1371/journal.pone.0319287)
Supplement: S6 File — S1 Table Quality appraisal summary. (DOCX) [file pone.0319287.s006.docx]

**Supplementary Information 6 – Quality Appraisal**

**Table S 1 Quality appraisal summary**

| **Reference** | **Study design** | **Where the methods used credible/ trustworthy?** | **How relevant is this document?** | **Other comments** |
| --- | --- | --- | --- | --- |
| Kirker et al. (1995) | Service Evaluation | 3 | 2 | Adds limited information to few MRTs |
| Brewin & Hazell (2004) | Service Evaluation | 5 | 5 | Adds to many MRTs |
| Main & Haig (2006) | Service Evaluation | 5 | 4 | Adds limited information to many MRTs |
| Bisiker & Millinchip (2007) | Service Evaluation | 4 | 3 | Adds limited information on a few MRTs |
| Townsend (2008) | Survey | 5 | 5 | Adds to many MRTs |
| Sweetland (2010) | Feasibility RCT | 5 | 5 | Adds and supports many MRTs |
| Royal College of Physicians (2011) | Service Evaluation | 5 | 3 | Adds limited information to few MRTs |
| Sweetland et al. (2014) | Case Study | 4 | 4 | Adds limited information to many MRTs |
| Jellie et al. (2014) | Qualitative Study | 5 | 5 | Adds limited information to many MRTs |
| McGregor (2014) | Service Evaluation | 5 | 4 | Adds information to many MRTs, |
| Ford (2020) | Protocol | 5 | 2 | Adds limited information to few MRTs |
| NICE (2022) | Guidelines | 5 | 2 | Adds limited information to few MRTs |
| NICE (2014) | Guidelines | 5 | 2 | Adds limited information to few MRTs |
| MRT: Middle-range theories; RCT: Randomised controlled trial; NICE: National Institute for Health and Care Excellence. | | | | |
