# Supplementary material for: Vocational rehabilitation for people with multiple sclerosis in the national health service of the United Kingdom: A realist evaluation
Source: PLoS One. 2025 Feb 25;20(2):e0319287. doi: 10.1371/journal.pone.0319287 (PMC11856266; doi:10.1371/journal.pone.0319287)
Supplement: Attachment — Submitted filename: PEER REVIEW OUTCOME_plosone.docx [file pone.0319287.s007.docx]

**Overview**

First, I commend the authors for the efforts and rigour invested into this study. This realistic evaluation sought to understand what VR interventions for people with MS delivered in the NHS of the UK work, under what circumstances, and why. The resulting programme theory illustrates a complex interplay between the healthcare and employment sectors to influence health and employment outcomes. VR programmes that offer timely support, tailored to the needs of the person with MS, and that support the employee beyond the healthcare context are most likely associated with improved employment outcomes. However, a number of revisions are required to put the manuscript in its best state.

**Areas of improvements**

The major areas of improvement include rearranging the objectives, the rough programme theory, streaming the definition of context to reflect the same concept in both the review and the interview arm and limitation. A few minor revisions are highlighted in the manuscript text.

**Objectives**

I would prefer that the specific objectives be re-arranged to ensure a systematism in their flow. I would think that “what works/context (VR interventions/resources and opportunities) should come first, then then the outcome and the mechanism that produced the outcome. That way, it remains in tune with CMO configuration. The present arrangement/flow is difficult to follow.

**Rough programme theory**

Whereas I acknowledge that the cited study [22] covers some of the facts outline in the rough programme theory, it does not do so completely. Hence, I would think that other relevant studies namely **1**. doi: 10.3233/WOR-210012. **2.** doi: 10.1177/1352458507078414 which speak into the fact of the rough programme theory should be cited. A theory is a collection of ideas/principles upon a practice/intervention may be based. Citing only self when there are other studies that speak into the fact of the rough programme theory makes the proposed theory weak. Besides, the more consistent the facts of a theory, the higher the application, utilization and universal transformation into a law/policy. In addition, I would prefer that the authors cite each of the facts presented in the rough programme. This will allow for confirmation of such facts in the cited studies. Also, it is difficult to tell which facts of the proposed theory is/could be measured at micro-level, meso and/or macro level. This should be clarified.

**Data Analysis**

This definition does not align with the context as highlighted in the rough programme theory. Here context referred to setting or stage. How was it different from the use of context in the rough programme theory? I think it would make sense to define the CMO and operationalize at their first appearance. Definition should be as consistent as possible in both the reviews and the qualitative arm/interviews.

**Limitations**

How does the variability in study characteristic contributes to the limitations?

**Verdict**

I consider the data provided in this manuscript essential given their relevance to subject of study. However, the manuscript requires some revisions to coherence and improve readability.
